# Joint modelling of potentially avoidable hospitalisation for five diseases accounting for spatiotemporal effects: A case study in New South Wales, Australia

**DOI:** 10.1371/journal.pone.0183653

**Published:** 2017-08-30

**Authors:** Jannah Baker, Nicole White, Kerrie Mengersen, Margaret Rolfe, Geoffrey G. Morgan

**Affiliations:** 1 Australian Research Council Centre of Excellence for Mathematical and Statistical Frontiers, Queensland University of Technology, Brisbane, Australia; 2 Cooperative Research Centres for Spatial Information, Melbourne, Australia; 3 The George Institute for Global Health, Sydney, Australia; 4 University Centre for Rural Health – North Coast, University of Sydney, Lismore, Australia; 5 School of Public Health, Faculty of Medicine, University of Sydney, Sydney, Australia; 6 North Coast Public Health Unit, Mid North Coast Local Health District, New South Wales, Australia; Johns Hopkins Bloomberg School of Public Health, UNITED STATES

## Abstract

**Background:**

Three variant formulations of a spatiotemporal shared component model are proposed that allow examination of changes in shared underlying factors over time.

**Methods:**

Models are evaluated within the context of a case study examining hospitalisation rates for five chronic diseases for residents of a regional area in New South Wales: type II diabetes mellitus (DMII), chronic obstructive pulmonary disease (COPD), coronary arterial disease (CAD), hypertension (HT) and congestive heart failure (CHF) between 2001–2006. These represent ambulatory care sensitive (ACS) conditions, often used as a proxy for avoidable hospitalisations. Using a selected model, the effects of socio-economic status (SES) as a shared component are estimated and temporal patterns in the influence of the residual shared spatial component are examined.

**Results:**

Choice of model depends upon the application. In the featured application, a model allowing for changing influence of the shared spatial component over time was found to have the best fit and was selected for further analyses. Hospitalisation rates were found to be increasing for COPD and DMII, decreasing for CHF and stable for CAD and HT. SES was substantively associated with hospitalisation rates, with differing degrees of influence for each disease. In general, most of the spatial variation in hospitalisation rates was explained by disease-specific spatial components, followed by the residual shared spatial component.

**Conclusion:**

Appropriate selection of a joint disease model allows for the examination of temporal patterns of disease outcomes and shared underlying spatial factors, and distinction between different shared spatial factors.

## 1. Background

The economic and social costs of the increasing incidence of potentially avoidable hospital admissions are a growing burden to health services in Australia and internationally [1–4]. In 1990, the concept of using ACS conditions as a proxy for avoidable hospital admissions was proposed [5]. In Australia, diabetes complications are the leading cause of avoidable hospitalisation, and together with COPD and angina due to CAD account for almost one half (44.5%) of all avoidable hospital admissions [6]. DMII accounts for 90–95% of all cases of diabetes [7–9] and has shown to be associated with other ACS conditions including COPD [10, 11], CAD [12–14], HT [15] and CHF [16, 17].

Residents of areas with indicators of socio-economic deprivation have been associated with increased risk of avoidable hospital admissions [18, 19] and emergency admissions after adjusting for other risk factors [20]. Individuals of lower SES are also more at risk for preventable hospitalisation even after adjusting for severity of illness [21]. To this end, accounting for area-level SES is important in joint disease spatiotemporal models exploring temporal trends in hospitalisation incidence for each ACS condition. Measures of SES are typically reported at a geographic residential scale such as a Statistical Local Area (SLA) or Local Government Area, so it is natural to consider this as a shared spatial factor. A shared spatial factor, or shared spatial component, is an underlying risk that is common to more than one disease. Although spatial studies have been performed in the US, UK and Europe to examine high-risk areas for avoidable hospitalisation, little is known about temporal trends in avoidable hospitalisation incidence in Australia specific to residential areas [20]. This paper aims to fill this gap in knowledge.

Much work in disease mapping has focused on the role of joint spatial modelling of two or more diseases [22–27]. Joint spatial modelling of diseases has the benefits of borrowing strength across both neighbouring regions *and* across diseases with common aetiological factors [28]. This is particularly useful when uncertainty is present due to sparse disease counts or under-reporting of disease [29, 30], as well as adjusting for unmeasured confounders [31, 32]. A shared component is defined as an underlying spatial component common to multiple diseases. The incorporation of shared and disease-specific spatial and temporal components has three key advantages. Firstly, it highlights regions with the greatest exposures to aetiological factors common to all diseases being modelled and shared temporal patterns in these disease outcomes: these regions would be expected to benefit most from additional resource placement and health promotion programmes to simultaneously decrease risk for multiple disease outcomes in one step. Secondly, it allows the exploration of differences between diseases with respect to spatial and temporal trends. Finally, it improves the precision of estimates compared with estimates from single disease models [22].

Early work in Bayesian joint disease modelling included the two-disease shared component model proposed by Knorr-Held and Best (2001) [22]. This model partitioned the relative risk of each of the two diseases into three spatial components allowing for spatial clustering: one component common to both diseases and two disease-specific components reflecting residual spatial variation in each disease. A variation on this model, presented by Dabney and Wakefield (2005), proposed one shared and one disease-specific component and was termed a “proportional model” [23]. Kim et al. (2001) proposed an alternative formulation using a bivariate Bayesian Poisson model to jointly model two diseases [24]. A shared spatial component model was extended to three or more diseases by Held et al. (2005) and was further developed by application to a four-disease case study accounting for spatial variation in smoking and alcohol consumption [25]. Further work by Held et al. (2006) that jointly modelled four infectious diseases, included three shared and four disease-specific spatial components to account for differential risk for diseases with related aetiological factors [30].

The incorporation of temporal effects was introduced by Richardson et al. (2006) in a two-disease model with disease-specific linear temporal effects and an exchangeable hierarchical structure for spatiotemporal interaction [26]. An alternative formulation was proposed by Tzala and Best (2008) for joint spatiotemporal modelling of three or more diseases [27]. The authors proposed a model with a shared spatial component specific to each year of the study period and a semi-parametric autocorrelation structure for time. Earnest et al. (2010) compared model fit between a standard shared component model to one with a zero-inflated Poisson extension to model excess zeros [29]. A recent study by Mahaki et al (2017) compared four alternative formulations for model fit, examining addition of a heterogeneity term and a shared spatiotemporal interaction term [33]. It is thus useful to compare formulations for several reasons: to examine the sensitivity to choice of hyperpriors [34]; and to examine which patterns of spatiotemporal interaction are appropriate to the dataset and which covariates should be included as shared components in the model [27]. Selection of the best-fitting formulation through model comparison has the potential to improve the accuracy and precision of risk estimates [33]. A detailed discussion of the relative advantages and disadvantaged of the above approaches is canvassed in Baker (2017) [35].

While semi-parametric temporal autocorrelation structures have been proposed in the literature, the inclusion of more structured temporal parameters may be useful when the focus is on comparing temporal trends in disease outcomes between diseases, on examining shared versus disease-specific temporal trends and on temporal prediction. Furthermore, it may be useful to examine whether the influence of underlying shared spatial factors may change over time for each disease. In addition, after adjusting for known underlying spatial factors common to all diseases studied, it may be useful to examine the effect of residual confounders not captured in the model and their influence on outcomes of each disease over time. These effects can be measured through the incorporation of a random error term to capture residual effects unexplained by variables in the model.

The motivation for this paper is to present a method for comparing temporal trends in disease outcomes between multiple diseases and examine the effect of residual shared latent factors over time after adjusting for known factors. The proposed methodology is applied to a case study that examines the incidence of hospitalisation for five ACS diseases in a regional area of New South Wales (NSW), Australia.

This paper has four objectives. First, to describe, implement and compare three variant formulations of a Bayesian shared component model allowing examination of temporal trends specific to each disease. Second, to examine disease-specific temporal trends in incidence of hospitalisation. Third, to distinguish shared and disease-specific spatial variation in hospitalisation incidence. The final objective is to examine geographic disparities in hospitalisation incidence for five ACS conditions.

## 2. Methods

### 2.1 Data

The data for the aforementioned case study was collected from two main sources. These sources are described in the subsections below.

#### 2.1.1 Admissions data

Data were routinely collected from all admissions among residents of the NSW region to any hospital in Australia between July 2001 and June 2006, representing five financial years of data. Residents belonged to one of 21 SLAs within the region, with an average estimated resident population (ERP) across SLAs of 21,000 (range 4,114 to 47,112). Hospitals in the dataset included urban and rural hospitals both within and outside the NSW region of residence, representing 100% coverage of hospital admissions within Australia among residents of the region. All private rural hospitals within the dataset were deidentified and aggregated into one category, thus the exact number of hospitals from which admissions records are included in the dataset is unknown.

Data were extracted for five ACS conditions: DMII, COPD, CAD, HT and CHF. Observed counts of hospital admissions for principal diagnosis of each of these five conditions were aggregated SLA of residence for each financial year using 2001 SLA codes. Using reported International Statistical Classification of Diseases-10 (ICD10) coding of principal diagnosis for each hospital admission, an ICD10 code between E11.0 and E11.99 inclusive was classified as a DMII admission. Similarly, a principal diagnosis of ICD10 code between J41 and J44 inclusive or J47 was classified as a COPD admission, between I20 and I25 inclusive as a CAD admission, between I10 and I15 inclusive with the exclusion of I11.0 as an HT admission, and I11.0 or I50 or J81 as a CHF admission.

#### 2.1.2 Demographic/Geographic information

Data were obtained from the Australian Bureau of Statistics (ABS) for ERP and Socio-Economic Indexes for Areas (SEIFA) in 2001 specific to each of the 21 SLAs of residence, based on information from the five-yearly Census [36, 37]. The SEIFA was developed by the ABS and ranks areas in Australia according to relative socio-economic advantage and disadvantage. Among the five financial years included in this analysis, ERP and SEIFA Indices at SLA level were only available for 2001, and 2001 estimates were used for all time periods in models described below. Four different measures of SES are available through the SEIFA: the Index of Relative Socio-Economic Advantage and Disadvantage (IRSAD), Index of Relative Socio-Economic Disadvantage (IRSD), Index of Education and Occupation (IEO) and the Index of Economic Resources (IER).

The IRSD includes only measures of relative disadvantage, including the proportion of residents with low income, lack of qualifications and working in unskilled occupations. The IRSAD includes measures of both relative advantage and disadvantage, including the proportion of residents with high and low income and working in skilled and unskilled occupations. The IER focuses on the financial aspects of relative socio-economic advantage and disadvantage, by summarising variables related to income and wealth. This includes the proportion of households with high or low income, the proportion of households paying low rent, and the proportion of owned homes. The IEO is designed to reflect the educational and occupational level of communities. The education variables include qualification levels achieved and whether further education is being undertaken. The occupation variables classify the workforce into skill levels and the unemployed.

For each of the four indices, a lower score was indicative of relative greater socio-economic deprivation. Within the dataset, the range for each measure of SES differed as follows. The range was 869–969 for IRSAD, 894–990 for IRSD, 857–946 for IER and 880–1017 for IEO. When split into quartiles, the lowest quartile of each range represents the greatest level of socio-economic deprivation, and the highest quartile represents the lowest level of deprivation.

#### 2.1.3 Exploratory analysis

Descriptive analysis of the dataset was performed, including an examination of hospitalisation rates for each disease in each financial year, and an assessment of the correlation between each SEIFA Index and hospitalisation rates for each disease. Radar plots of SEIFA Index quartiles and hospitalisation rate quartiles were constructed and evaluated, as well as maps of SEIFA Index quartiles compared with hospitalisation rate quartiles for each SLA within the region.

### 2.2 Fundamental models

Three Bayesian spatiotemporal shared component formulations were compared for the estimation of temporal trends in hospitalisation rates for each of the five ACS conditions described above. Time is measured in financial years; a “year” is defined as a financial year for the purposes of this paper. The first two models, Models A and B, examine whether the influence of shared underlying spatial factors changes over time by allowing different factor loadings for each disease for each of the five years in the analysis. Model A imposes a linear structure upon the relationship between year and the log hospitalisation rate for each disease; Model B extends on Model A by including both shared and disease-specific linear temporal components. Model C is a simplification of Model A and includes factor loadings common across all years for the shared underlying spatial factors for each disease.

Markov chain Monte Carlo (MCMC) simulations were used to model joint patterns of hospitalisation rates for the five conditions studied. This was undertaken using R 3.1.2 and WinBUGS 14 [38, 39]. The R2WinBUGS package in R 3.1.2 was used to import data, specify parameter names, initial values and model specifications, and to invoke a BUGS model run in WinBUGS 14. Each model was run for a total of 200,000 iterations with the first 90,000 iterations discarded as the burnin period. For its association with each outcome, a covariate was identified as important if the 95% credible interval (CI) for its associated coefficient did not cover zero. Similarly, an SLA was flagged as having differential excess risk if the 95% CI for its associated random effect did not cover zero. A high risk area had a substantively greater risk of hospitalisation relative to other areas.

Formulations for the three compared models are described below.

#### 2.2.1 Model A: Disease-specific temporal trends

We assign each disease and each year an index as follows: DMII (*j* = 1), COPD (*j* = 2), CAD (*j* = 3), HT (*j* = 4) and CHF (*j* = 5); *k* = 1, …, 5 for 2001–2005. For SLA of residence, *i* = 1, …, 21, for disease, *j* = 1, …, 5 and for year, *k* = 1, …, 5, the observed number of hospital admissions *Y*_*ijk*_ with a principal diagnosis for disease *j* is modelled as a Poisson generalised linear mixed model (GLMM) with a shared spatial component *s*_*i*_ weighted by factor *δ*_*jk*_ for each disease in each year, a disease-specific spatial component *v*_*ij*_, and a temporal component *β*_*j*_*t*_*k*_ where *β*_*j*_ is a disease-specific coefficient for the year of admission, *t*_*k*_. Let the ERP for SLA *i* using 2001 estimates be denoted *n*_*i*_, and *θ*_*ijk*_ denote the estimated hospitalisation incidence rate for SLA *i* for disease *j* in year *k*.

A sum-to-zero constraint is placed on the sum of the log of weighting factors for the shared component at each time point. This is an extension of the joint multiple disease model with sum-to-zero constraints for one time point as described by Held et al. (2005). The shared and disease-specific spatial components are assigned conditional autoregressive (CAR) priors as first described by Besag, York and Mollie (1991) [40]. A CAR specification was selected due to the presence of sparse data for smaller SLAs, motivating the use of local smoothing to borrow strength across neighbouring areas.

Let *α*_*j*_ denote a disease-specific intercept, *u*_*ijk*_ the uncorrelated residual error for each observation and *σ*^2^ the variance for the log of *θ*_*ijk*_. We define *I* = 21, *J* = 5 and *K* = 5. The model takes the following formulation:

$$Y_{ijk}\sim Poisson\left(n_{i}\theta_{ijk}\right)$$

$$\text{log}\left(\theta_{ijk}\right)\sim N\left(s_{i}\delta_{jk}+\nu_{ij}+\beta_{j}t_{k}+\alpha_{j}+u_{ijk},\sigma^{2}\right)$$ (1)

$$\overset{J}{\underset{j=1}{\sum }}\text{log}\left(\delta_{jk}\right)=0$$

Priors for hyperparameters in the model are as follows:

$$u_{ijk}\sim N\left(0,\omega_{j}^{2}\right)$$ (2)

$$s_{i}|s_{\left(-i\right)},\sigma_{s}^{2}\;\sim N\left(\frac{\sum_{h=1}^{n}s_{h}w_{hi}}{m_{i}},\frac{\sigma_{s}^{2}}{m_{i}}\right)$$

$$v_{ij}|v_{\left(-i\right)j},\sigma_{vj}^{2}\;\sim N\left(\frac{\sum_{h=1}^{n}v_{hj}w_{hi}}{m_{i}},\frac{\sigma_{vj}^{2}}{m_{i}}\right)$$

$$w_{hi}=\{\begin{array}{ll} 1 & if\;h\;and\;i\;are\;neighbours\\ 0 & otherwise \end{array}$$

for *i* = 1, …, 21 with (−*i*) denoting all regions excluding *i*. $\frac{\sum_{h=1}^{n}s_{h}w_{hi}}{m_{i}}$ is the average correlated random effect for the neighbours of region *i*, *m*_*i*_ is the number of such neighbours, and $\sigma_{s}^{2}$ is the overall spatial variance across all regions. A neighbour is defined as any region immediately adjacent in space to region *i*. It can be seen that this type of prior induces a form of local smoothing across regions, where the degree of smoothing is controlled by the spatial correlation between regions.

Additional priors for hyperparameters include Normal distributions for *log*(*δ*_*jk*_), *α*_*j*_ and *β*_*j*_; *log*(*δ*_*jk*_)~N(0,0.25); *α*_*j*_, *β*_*j*_ ~ *N*(0,100)), and $\sigma_{\text{S}}^{2}$, $\sigma_{Vj}^{2}$, *σ*^2^, $\omega_{j}^{2}\sim IG\left(1.0,0.01\right)$. The selected prior for *log*(*δ*_*jk*_) was based on the prior belief that *log*(*δ*_*jk*_) is between -0.5 and 0.5 with 95% probability for each disease at each timepoint, similar to foundational shared component models in the literature [22, 25]. Given the lack of prior knowledge surrounding estimates for other parameters in the model, priors for other parameters were selected as they are uninformative while allowing a wide range of plausible values.

#### 2.2.2 Model B: Shared and disease-specific temporal trends

Model B is an extension of Model A that includes coefficients for both shared and disease-specific temporal components, in order to examine evidence for a temporal trend common to all diseases. For Model B, Eq (1) is changed to:

$$\text{log}\left(\theta_{ijk}\right)\sim N(s_{i}\delta_{jk}+\nu_{ij}+\left(\beta_{0}+\beta_{j})t_{k}+\alpha_{j}+u_{ijk},\sigma^{2}\right)$$ (3)

Given *β*_*o*_, the interpretation of *β*_*j*_ has now changed to the difference between each disease-specific temporal trend and the shared temporal trend common to all diseases in the model. Priors for hyperparameters are as described for Model A, and *N*(0,100) for the new parameter, *β*_0_. As described above, priors were selected to be uninformative and allow a wide range of plausible values.

#### 2.2.3 Model C: Shared weighting factor

Model C is a simplification of Model A. The year-specific weighting factor *δ*_*jk*_ is changed to a single weighting factor *δ*_*j*_ for each disease, *j*, across all years as follows:

$$\text{log}\left(\theta_{ijk}\right)\sim N\left(s_{i}\delta_{j}+\nu_{ij}+\beta_{j}t_{k}+\alpha_{j}+u_{ijk},\sigma^{2}\right)$$ (4)

$$\overset{J}{\underset{j=1}{\sum }}\text{log}\left(\delta_{j}\right)=0$$

Priors for hyperparameters are as described for Model A in Eq (2), and *log*(*δ*_*j*_)~*N*(0,0.25).

### 2.3 Model comparison

Model fit was compared between the three basic models described above using Deviance Information Criterion (DIC), the log likelihood, root mean squared error and predictive concordance. Each measure is briefly described in S1 Text.

### 2.4 Sensitivity analysis

Sensitivity analysis was performed to determine the robustness of inferences from the selected model. Different classes of prior distributions were fit and results were compared. The selected model from Part 2.4 was rerun with the following variations of prior distribution:

1. $\omega_{j}^{2},\sigma^{2},\sigma_{\text{s}}^{2},\sigma_{vj}^{2}\sim IG\left(1.0,0.01\right)$—Base model
2. $\omega_{j}^{2},\sigma^{2},\sigma_{\text{s}}^{2},\sigma_{vj}^{2}\sim IG\left(0.001,0.001\right)$
3. $\omega_{j}^{2},\sigma^{2},\sigma_{\text{s}}^{2},\sigma_{vj}^{2}\sim IG\left(0.5,0.0005\right)$
4. *ω*_*j*_, *σ*, *σ*_*s*_, *σ*_*vj*_~*Uniform*(0,5)
5. *ω*_*j*_, *σ*, *σ*_*s*_, *σ*_*vj*_~*N*(0,1)*I*(0,∞)
6. *log*(*ω*_*j*_), *log*(*σ*), *log*(*σ*_*s*_), *log*(*σ*_*vj*_)~*N*(0,0.25)

For each model included in sensitivity analysis, the posterior mean estimates and 95% CIs for model parameters were compared to check for model robustness. The four types of priors above represent a wide variety of classes of distribution, with differing means, variances and probabilities assigned to each value within the distribution. Thus, if similar estimates are obtained despite the choice of prior, this indicates that inferences are robust.

Following sensitivity analysis, the selected model was utilised to examine temporal patterns in hospitalisation rates for each disease, the influence of SES, temporal patterns in the influence of the residual shared spatial component after accounting for SES, and the proportion of variation in hospitalisation rates that is explained by each spatial component.

## 3. Results

### 3.1 Data

**Fig 1:**
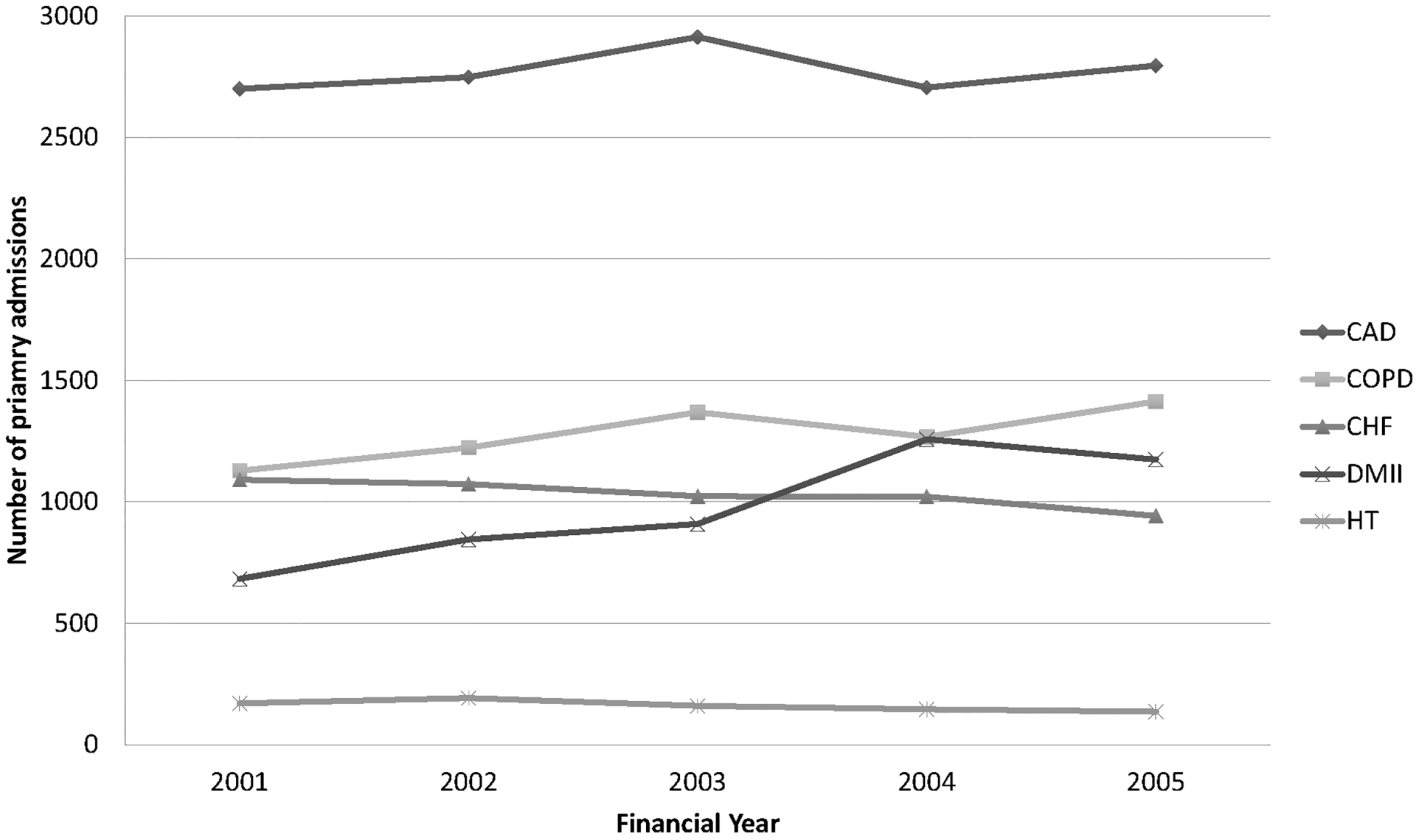
Temporal patterns in observed numbers of primary admissions for five diseases across a regional area of New South Wales. CAD = coronary arterial disease, COPD = chronic obstructive pulmonary disease, CHF = congestive heart failure, DMII = type II diabetes mellitus, HT = hypertension.

**Fig 2:**
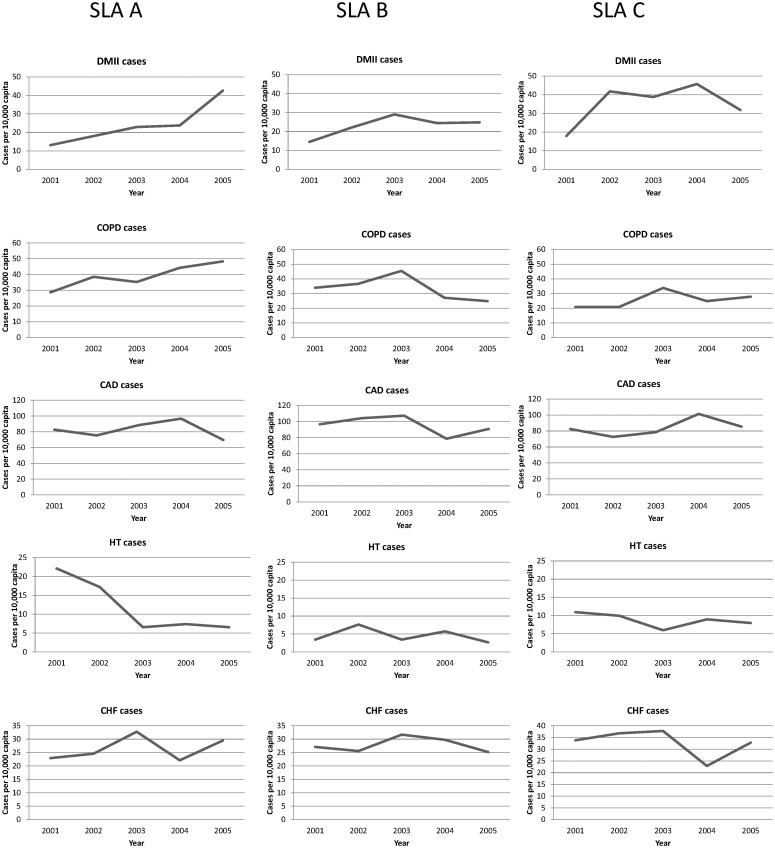
Temporal patterns in number of hospital admissions per 10,000 capita for three randomly selected New South Wales Statistical Local Areas for five diseases. DMII = type II diabetes mellitus, COPD = chronic obstructive pulmonary disease, CAD = coronary arterial disease, HT = hypertension, CHF = congestive heart failure.

Over the study period, among the five ACS conditions included in this analysis, hospitalisation was most frequent for CAD, followed by COPD, CHF, DMII and HT respectively. Among the estimated 2001 ERP of 443,199 across the region, there were a recorded 13,866 cases of CAD, 6,401 cases of COPD, 5150 cases of CHF, 4,869 cases of DMII and 804 cases of HT principal hospital admissions over the five-year study period. Temporal patterns in number of admissions for each disease in this region are shown in Fig 1. Overall, the number of admissions for DMII and COPD appears to be increasing over time, and the number of admissions for CHF appears to be decreasing over time. Numbers of hospital admissions for CAD and HT remained relatively stable over the study period. From exploratory analysis, there appear to be varying temporal patterns for hospitalisation for each disease by area of residence, especially for DMII, COPD and HT. Fig 2 shows temporal patterns in the number of admissions per 10,000 capita for each disease for three randomly selected SLAs. Whereas for some SLAs, the number of admissions per 10,000 capita appears to be consistently increasing, decreasing or remaining similar across the study period, a nonlinear pattern is observed for other SLAs in the region. The ERP and age-gender composition of the background population in each area remains relatively stable over the study period based on 2001 and 2006 Census statistics. Therefore, it is possible that differences in temporal patterns between areas may be related to differences in underlying aetiological factors between areas and may change over time, the investigation of which is an objective of this study.

Table 1 summarises Pearson correlations between the four SES measures and overall hospitalisation rate per 10,000 capita across the 21 SLAs for each disease across the study period. All four measures of SES are negatively correlated with hospitalisation rates for all five diseases. Thus areas that are less deprived tend to have lower rates of hospitalisation for ACS conditions. In general, IRSAD and IEO Indexes have the strongest association with hospitalisation rates across all diseases, indicated by the size of the Pearson’s correlations and associated p values (Table 1). This suggests that variation in overall relative advantage and disadvantage, education and occupation may play an important part in explaining variation in hospitalisation rates. Among the five ACS conditions, CHF appears to have the strongest correlation with SES measures, followed by HT, COPD, CAD and DMII respectively.

**Table 1:** Pearson’s correlations between incidence of hospitalisation for each of five diseases across the study period and measures of socio-economic status across Statistical Local Areas within the regional area of New South Wales.

	DMII	COPD	CAD	HT	CHF
IRSAD	-0.20	-0.24	-0.18	-0.30	-0.32
IRSD	-0.11	-0.10	-0.09	-0.20	-0.34
IER	-0.12	-0.21	-0.05	-0.31	-0.21
IEO	-0.23	-0.24	-0.24	-0.26	-0.32

DMII = diabetes mellitus type II, COPD = chronic obstructive pulmonary disease, CAD = coronary arterial disease, HT = hypertension, CHF = congestive heart failure, IRSAD = Index of Relative Socio-Economic Advantage and Disadvantage, IRSD = Index of Relative Socio-Economic Disadvantage, IER = Index of Economic Resources, IEO = Index of Education and Occupation

**Fig 3:**
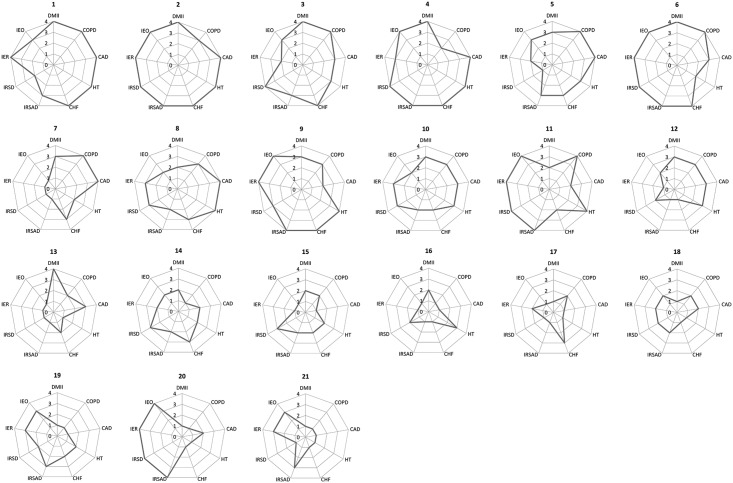
Radar plots of quartiles for incidence of hospitalisation for each of five diseases across the study period (4 = highest incidence, 1 = lowest incidence) and quartiles of socio-economic deprivation measured by four Indexes (4 = most deprived, 1 = least deprived) across the Statistical Local Areas within the regional area of New South Wales. Statistical Local Areas are ordered from highest to lowest total incidence of hospitalisation across all diseases. DMII = diabetes mellitus type II, COPD = chronic obstructive pulmonary disease, CAD = coronary arterial disease, HT = hypertension, CHF = congestive heart failure, IRSAD = Index of Relative Socio-Economic Advantage and Disadvantage, IRSD = Index of Relative Socio-Economic Disadvantage, IER = Index of Economic Resources, IEO = Index of Education and Occupation.

Fig 3 shows radar plots for each of the 21 SLAs. Quartiles internal to the dataset are plotted for hospitalisation rate for each disease and deprivation level as measured by IRSAD, IRSD, IER and IEO indexes. Quartile 4 indicates the largest hospitalisation rates and largest deprivation levels among the 21 SLAs, and quartile 1 indicates the smallest hospitalisation rates and least deprived areas. It is clear from the radar plots that areas with large hospitalisation rates tend to have greater levels of socioeconomic deprivation, and areas with small hospitalisation rates tend to have less deprivation. Results of this exploratory analysis support the inclusion of SES measures within the joint disease model as an additional shared spatial component common to all diseases.

### 3.2 Model comparison

All models were assessed to have converged and similar estimates were produced for disease-specific temporal trends from all three models. Results from Models A and B indicate substantive variation in disease-specific factor loadings for the shared spatial component over time. The term for the shared temporal component in Model B was not substantive.

Table 2 shows results of comparison of Models A-C for each disease. Overall, Models A and B performed equally well on all measures of fit and better than Model C based on DIC and log likelihood. This suggests that the influence of shared underlying aetiological factors common to all five diseases may have changed over time, and is important to account for within this application. Across all models, the best fit was observed for HT and the poorest fit for CAD based on log likelihood and RMSE; however, observed values for HT fell into predicted 95% CIs less frequently as measured by predictive concordance.

**Table 2:** Goodness of fit comparisons between basic joint disease models.

	Model A	Model B	Model C
DIC	3690.76	3690.12	3788.06
Log likelihood			
DMII	-336.3	-336.1	-337.3
COPD	-360.0	-361.0	-360.2
CAD	-394.5	-394.6	-394.8
HT	-248.7	-248.0	-250.4
CHF	-345.4	-345.9	-356.6
Overall	-1685	-1686	-1699
RMSE			
DMII	9.65	9.64	9.62
COPD	11.06	11.08	11.03
CAD	16.12	16.10	16.17
HT	4.00	3.99	3.94
CHF	9.83	9.83	9.88
Overall	10.87	10.86	10.88
Predictive concordance (95% CI)			
DMII	1.00	1.00	0.99
COPD	0.98	0.98	0.98
CAD	0.99	0.99	0.99
HT	0.89	0.88	0.89
CHF	0.99	0.98	0.96
Overall	0.97	0.97	0.96

DIC = Deviance Information Criteria, DMII = diabetes mellitus type II, COPD = chronic obstructive pulmonary disease, CAD = coronary arterial disease, HT = hypertension, CHF = congestive heart failure, RMSE = root mean squared error, CI = credible interval. “Overall” refers to the overall model. Lower values of DIC and RMSE and higher values of log likelihood and predictive concordance indicate a better fit.

Models A and B performed equally well on posterior predictive checks, however, the shared temporal component in Model B was found to be negligible. Therefore, Model A was selected as the marginally preferred model for further analysis.

As a comparison to Model A, a model with dummy variables for year was also fit which did not constrain the temporal component to be linear. Coefficients for each year from this model followed a roughly linear trend for each disease matching findings from Model A, thus supporting the use of a linear temporal trend.

## 4. Extended model

Model A was extended to include an additional component to account for the influence of SES on hospitalisation rates. Only one of the four SES measures was included in the model at each time, and resulting estimates for unknown parameters were compared. The formulation for this extended model is as follows:

$$Y_{ijk}\sim Poisson\left(n_{ijk}\theta_{ijk}\right)$$

$$\text{log}\left(\theta_{ijk}\right)\sim N\left(s_{i}\delta_{jk}+\left(\phi +\gamma_{j}\right)x_{i}+\nu_{ij}+\beta_{j}t_{k}+\alpha_{j}+u_{ijk},\sigma^{2}\right)$$ (5)

where *x*_*i*_ is the region-specific SEIFA score for one of four measures: IRSAD, IRSD, IEO and IER, *ϕ* is the mean random effect for the SEIFA index and *γ*_*j*_ is the disease-specific variation from this mean random effect for each disease *j*. SEIFA scores were included as a continuous measure in order to estimate the influence of each unit increase on log(*θ*_*ijk*_). Priors for hyperparameters in the model are as described for Model A in Eq (2), and *N*(0,10) for *γ*_*j*_ and *N*(0,100) for *θ*, allowing a wide range of plausible values. A sum-to-zero constraint was applied to *γ*_*j*_.

Table 3 compares the estimated coefficients from each of the four SES models. The shared SES component for the SEIFA Index, *ϕ*, was found to be associated with hospitalisation rates for IRSAD, IER and IEO but not for IRSD. Among IRSAD, IER and IEO models, the estimated mean and 95% CI for *ϕ* was similar for each of these three measures across all included diseases. There was no evidence of substantive disease-specific variation from ∅ (measured by *γ*_*j*_) for any of the five diseases. The DIC was similar for these three models indicating similar goodness of fit. As little difference was found in the estimated effect of IRSAD, IER and IEO on hospitalisation rates, IRSAD was selected to be included in further analysis.

**Table 3:** Comparison of estimated coefficients from joint disease models incorporating measures of socio-economic status as an additional shared spatial component. Coefficients are summarised with regard to posterior mean and 95% credible interval.

Model	IRSAD	IRSD	IER	IEO
*ϕ*	-0.008 (-0.012, -0.004)	-0.002 (-0.007, 0.003)	-0.008 (-0.013, -0.003)	-0.007 (-0.010, -0.004)
*γ*_1_	0.000 (-0.006, 0.007)	0.000 (-0.007, 0.010)	0.000 (-0.007, 0.008)	0.000 (-0.005, 0.006)
*γ*_2_	0.002 (-0.004, 0.009)	0.004 (-0.004, 0.013)	-0.000 (-0.000, 0.008)	0.002 (-0.003, 0.007)
*γ*_3_	0.002 (-0.005, 0.009)	0.000 (-0.008, 0.009)	0.005 (-0.004, 0.013)	0.000 (-0.005, 0.006)
*γ*_4_	-0.009 (-0.017, -0.000)	-0.008 (-0.020, 0.003)	0.009 (-0.018, 0.000)	-0.006 (-0.013, 0.000)
*γ*_5_	0.004 (-0.001, 0.010)	0.003 (-0.005, 0.009)	0.004 (-0.002, 0.010)	0.003 (-0.000, 0.008)
DIC	3692.760	3689.370	3694.410	3692.900

IRSAD = Index of Relative Socio-Economic Advantage and Disadvantage, IRSD = Index of Relative Socio-Economic Disadvantage, IER = Index of Economic Resources, IEO = Index of Education and Occupation. *ϕ* is the shared component associated with each measure of socio-economic status (IRSAD, IRSD, IER and IEO). *γ*_1–5_ is the disease-specific factor loading associated with *ϕ* for type II diabetes mellitus, chronic obstructive pulmonary disease, coronary arterial disease, hypertension and congestive heart failure respectively.

### 4.1 Sensitivity analysis

Similar parameter estimates were obtained from models included in sensitivity analysis following the model formulation with IRSAD as the measure of SES. However the DIC for each model varied slightly. The DIC for each model within the sensitivity analysis is summarised in Table 4. Overall, the model with uniform priors for *σ*_*s*_, *σ*_*vj*_, *σ* and *ω*_*j*_ had the smallest DIC (3674.06 vs. 3692.76 for the base model) indicating the best fit. Thus, results of our final model are presented from the model with uniform priors.

**Table 4:** Comparison of goodness of fit of models included in sensitivity analysis.

Variation	Hyperparameters	Priors	DIC
1	$\sigma_{\text{S}}^{2}$, $\sigma_{Vj}^{2}$, *σ*^2^, $\omega_{j}^{2}$	*IG*(1.0,0.01)	3692.760
2	$\sigma_{\text{S}}^{2}$, $\sigma_{Vj}^{2}$, *σ*^2^, $\omega_{j}^{2}$	*IG*(0.001,0.001)	3685.160
3	$\sigma_{\text{S}}^{2}$, $\sigma_{Vj}^{2}$, *σ*^2^, $\omega_{j}^{2}$	*IG*(0.5,0.0005)	3692.340
4	*σ*_*S*_, *σ*_*Vj*_, *σ*, *ω*_*j*_	*Uni*(0,5)	3674.060
5	*σ*_*S*_, *σ*_*Vj*_, *σ*, *ω*_*j*_	*N*(0,1)*I*(0,∞)	3700.160
6	ln(*σ*_*S*_), ln(*σ*_*Vj*_), ln(*σ*), ln(*ω*_*j*_)	*N*(0,0.25)	3687.110

DIC = Deviance Information Criteria

Table 5 summarises the estimated coefficients from the final model. The mean hospitalisation rate per 10,000 capita for each disease across the study period is estimated by $10,000e^{\alpha_{j}}$. Consistent with descriptive plots, Figs 1 and 2, the estimated rates are highest for CAD, followed by COPD, CHF, DMII and HT respectively.

**Table 5:** Estimated parameters from the selected joint disease model. Coefficients are summarised with regard to posterior mean and 95% credible interval.

Disease *j*	1 (DMII)	2 (COPD)	3 (CAD)	4 (HT)	5 (CHF)
*α*_*j*_	-6.199 (-6.26, -6.141)	-5.922 (-5.970, -5.876)	-5.212 (-5.243, -5.182)	-7.990 (-8.091, -7.891)	-6.115 (-6.161, -6.071)
exp(*α*_*j*_)*10,000	20 (19–22)	27 (26–28)	55 (53–56)	3 (3–4)	22 (21–23)
*β*_*j*_	0.152 (0.113, 0.194)	0.045 (0.014, 0.077)	0.005 (-0.016, 0.025)	-0.064 (-0.131, -0.000)	-0.035 (-0.065, -0.009)
*σ*	0.066 (0.002, 0.121)				
*ω*_*j*_	0.211 (0.137, 0.286)	0.147 (0.060, 0.217)	0.057 (0.004, 0.118)	0.242 (0.099, 0.368)	0.085 (0.006, 0.159)
*σ*_*S*_	0.852 (0.581, 1.250)				
*σ*_*Vj*_	1.008 (0.648, 1.517)	0.990 (0.658, 1.460)	1.051 (0.730, 1.536)	1.103 (0.728, 1.666)	0.863 (0.560, 1.293)
*ϕ*	-0.008 (-0.013, -0.003)				
*γ*_*j*_	0.000 (-0.006, 0.008)	0.002 (-0.006, 0.010)	0.001 (-0.006, 0.009)	-0.008 (-0.017, 0.002)	0.003 (-0.003, 0.011)

DMII = diabetes mellitus type II, COPD = chronic obstructive pulmonary disease, CAD = coronary arterial disease, HT = hypertension, CHF = congestive heart failure

Consistent with appearances in Fig 1, the model estimates an overall increase over time in hospitalisation rates for DMII and COPD, and a decrease for CHF as measured by the disease-specific temporal coefficient *β*_*j*_. Hospitalisation rates did not substantively change over time for CAD nor HT over the study period. The largest variation in hospitalisation rates across areas after accounting for temporal trends and effect of shared spatial components, was seen for HT and the lowest for CAD as measured by the standard deviation for correlated residual error (*ω*_*j*_). Compared with variation in the shared spatial component measured by *σ*_*S*_, variation in disease-specific spatial components measured by *σ*_*Vj*_ was greater. Similarly, compared with the common term for uncorrelated residual error, *σ*, the variation in disease-specific uncorrelated error measured by *ω*_*j*_ is greater.

Tables 6 and 7 summarise the posterior coefficients and proportion of overall spatial variation that is explained by each of three components for each disease. The first component is the shared SES component weighted by its disease-specific factor loading, the second is the residual shared spatial component after accounting for the influence of SES, weighted by its disease-specific factor loading specific to each year, and the third is the disease-specific spatial component. The variation in each of these three components is measured by its variance across SLAs. The variance from the SES component measured by IRSAD Index was small for all diseases, ranging from 0.03 for CAD to 0.24 for HT. This indicates that hospitalisation for CAD has the weakest and for HT has the strongest association with SES. In general, spatial variation explained by the residual shared spatial component was smaller than variation explained by the disease-specific spatial component across all years with the exception of 2004. In 2004, there was a decrease in disease-specific factor loading estimates and in the spatial variation explained by the residual shared component across all diseases. A similar pattern was observed in results from basic models A and B, all SES models and models included in the sensitivity analysis. Thus the proportion of spatial variation explained by each of the three components above varied both between diseases and across years (Table 7).

**Table 6:** The estimated coefficients for of spatial variation in incidence of hospitalisation for five diseases that is explained by variation in: (a) the shared socio-economic component weighted by its disease-specific factor loading, (b) the residual shared spatial component after accounting for the influence of (a), weighted by its disease-specific factor loading specific to each year and (c) the disease-specific spatial component, at each time point *k* for each disease *j*. Coefficients are summarised with regard to posterior mean and 95% credible interval.

Disease *j*	1 (DMII)	2 (COPD)	3 (CAD)	4 (HT)	5 (CHF)
Variation in SES component	0.06 (0.00,0.22)	0.05 (0.00,0.24)	0.06 (0.00,0.21)	0.24 (0.03,0.61)	0.03 (0.00,0.13)
Variation from residual shared spatial component, $\sigma_{\text{S}}^{2}$	0.65 (0.34, 1.56)				
Factor loading, *δ*_*jk*_, by year					
2001	1.48 (1.20,1.78)	1.44 (1.20,1.69)	1.32 (1.17,1.49)	1.27 (0.83,1.74)	1.36 (1.15,1.61)
2002	1.40 (1.14,1.68)	1.39 (1.14,1.63)	1.28 (1.14,1.47)	1.14 (0.70,1.62)	1.39 (1.19,1.62)
2003	1.51 (1.23, 1.79)	1.58 (1.34,1.86)	1.33 (1.18,1.50)	1.46 (1.08,1.88)	1.39 (1.19,1.61)
2004	0.24 (0.14,0.35)	0.20 (0.12,0.29)	0.36 (0.25,0.47)	0.43 (0.19,0.75)	0.26 (0.15,0.37)
2005	1.39 (1.12,1.66)	1.70 (1.44,1.99)	1.26 (1.11,1.43)	1.23 (0.79,1.66)	1.55 (1.32,1.80)
Variation from weighted residual spatial component					
2001	0.64 (0.38,1.04)	0.60 (0.36,0.88)	0.51 (0.34,0.75)	0.48 (0.18,0.84)	0.54 (0.34,0.79)
2002	0.57 (0.33,0.92)	0.56 (0.32,0.85)	0.48 (0.32,0.69)	0.40 (0.11, 0.81)	0.56 (0.34,0.80)
2003	0.66 (0.36,1.00)	0.72 (0.45,1.04)	0.51 (0.35,0.72)	0.63 (0.32,1.12)	0.56 (0.35,0.82)
2004	0.02 (0.00,0.04)	0.01 (0.00,0.03)	0.04 (0.01,0.08)	0.06 (0.01,0.14)	0.02 (0.01,0.05)
2005	0.57 (0.31,0.88)	0.83 (0.53,1.14)	0.46 (0.30,0.67)	0.46 (0.15,0.86)	0.70 (0.45,0.97)
Disease-specific spatial variation, $\sigma_{Vj}^{2}$	0.88 (0.42,2.30)	0.87 (0.43,2.13)	1.00 (0.53,2.36)	1.07 (0.53,2.77)	0.65 (0.31,1.67)

SES = socio-economic status measured by the Index of Relative Socio-Economic Advantage and Disadvantage, DMII = diabetes mellitus type II, COPD = chronic obstructive pulmonary disease, CAD = coronary arterial disease, HT = hypertension, CHF = congestive heart failure.

**Table 7:** The estimated proportion of spatial variation in incidence of hospitalisation for five diseases that is explained by variation in: (a) the shared socio-economic component weighted by its disease-specific factor loading, (b) the residual shared spatial component after accounting for the influence of (a), weighted by its disease-specific factor loading specific to each year and (c) the disease-specific spatial component, at each time point *k* for each disease *j*. Proportions are summarised with regard to posterior mean and 95% credible interval.

Disease *j*	1 (DMII)	2 (COPD)	3 (CAD)	4 (HT)	5 (CHF)
Proportion of spatial variation attributed to SES component					
2001	4% (0–12%)	3% (0–12%)	4% (0–12%)	13% (1–31%)	3% (0–9%)
2002	4% (0–13%)	3% (0–12%)	4% (0–12%)	13% (1–31%)	3% (0–9%)
2003	4% (0–12%)	3% (0–11%)	4% (0–12%)	12% (1–29%)	3% (0–9%)
2004	6% (0–20%)	5% (0–19%)	5% (0–17%)	17% (2–38%)	4% (0–16%)
2005	4% (0–13%)	3% (0–11%)	4% (0–12%)	13% (1–31%)	2% (0–8%)
Proportion of spatial variation from residual shared component					
2001	38% (21–57%)	38% (21–55%)	31% (17–46%)	25% (10–43%)	42% (24–60%)
2002	35% (19–54%)	36% (20–53%)	30% (16–45%)	21% (7–39%)	42% (25–61%)
2003	39% (21–58%)	42% (25–59%)	31% (17–47%)	30% (15–49%)	43% (25–61%)
2004	2% (0–5%)	1% (0–4%)	3% (1–8%)	4% (1–11%)	3% (1–7%)
2005	35% (19–53%)	45% (27–62%)	29% (15–44%)	24% (8–42%)	48% (29–66%)
Proportion of spatial variation that is disease-specific					
2001	58% (39–77%)	59% (41–77%)	65% (49–81%)	62% (41–82%)	56% (38–74%)
2002	61% (41–79%)	61% (43–79%)	66% (50–82%)	65% (42–87%)	55% (37–73%)
2003	58% (38–77%)	55% (38–73%)	65% (49–81%)	58% (37–79%)	55% (37–73%)
2004	92% (78–99%)	94% (80–99%)	92% (79–98%)	79% (56–97%)	93% (81–99%)
2005	61% (42–79%)	52% (35–70%)	67% (51–82%)	63% (41–84%)	50% (32–69%)

SES = socio-economic status measured by the Index of Relative Socio-Economic Advantage and Disadvantage.

**Fig 4:**
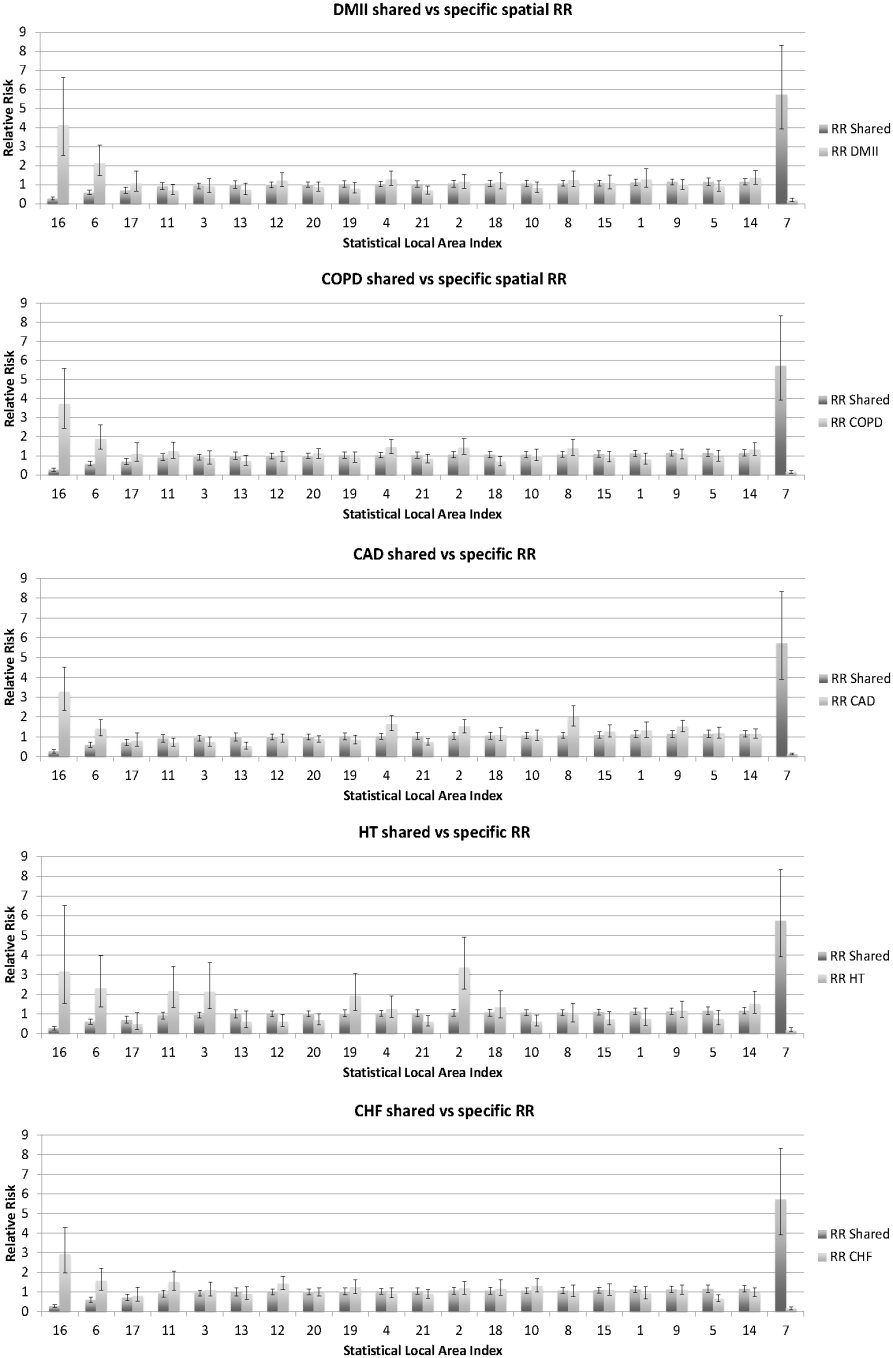
Comparison of mean estimates of relative risk for hospitalisation for five diseases, separated into shared and disease-specific spatial components across Statistical Local Areas within the regional area of New South Wales from the selected model. Error bars represent 95% credible intervals for estimates. Statistical Local Areas 1–21 are ordered by the size of the estimated relative risk associated with their shared component, from smallest to largest. RR = relative risk, DMII = diabetes mellitus type II, COPD = chronic obstructive pulmonary disease, CAD = coronary arterial disease, HT = hypertension, CHF = congestive heart failure.

Fig 4 compares the relative risk of the residual shared and disease-specific spatial components for each disease, with SLAs 1–21 ordered by the size of the relative risk associated with their shared component. Areas with small values for the shared spatial component tended to have large values from the disease-specific spatial component and vice-versa.

## 5. Discussion

We have proposed three Bayesian shared component model formulations to study shared and disease-specific spatial and temporal trends in hospitalisation rates for five ambulatory care sensitive conditions. Extending upon the two-disease and multiple disease spatiotemporal joint disease formulations proposed by Richardson et al. (2006) and Tzala and Best (2008), advantages of our selected model include:

1. The disease- and time-specific factor loadings for the shared spatial component allow examination of changes in underlying shared factors over time
2. The log-linear structure of the disease-specific temporal components is useful for prediction of disease counts specific to area and time and can be extrapolated to timepoints external to the dataset
3. A shared temporal component can be added to the model where appropriate for the application, to investigate temporal trends common to all diseases in the study based on changes over time in shared underlying aetiological factors
4. Where appropriate to the application, the model can be simplified to include disease-specific factor loadings for the shared spatial component common to all timepoints where there is no evidence of change in underlying shared factors over time
5. Where appropriate to the application, additional shared spatial components can be added to the model such as we have demonstrated by adding an additional shared component for SES. This allows spatial and temporal patterns for different underlying aetiological factors to be distinguished from each other, and allows the exploration of patterns of residual shared and disease-specific spatial factors after accounting for factors that are known to be shared by all or some of the diseases in the study.

The five conditions included in this study were selected as several share common aetiological risk factors. All five diseases share smoking history and increased age as underlying risk factors [10, 13]. In addition, risk factors shared between DMII, CAD and CHF include physical inactivity, obesity and central obesity, hypertension, and raised concentrations of insulin, HbA1c, triglycerides and fasting plasma glucose [12, 16, 17].

COPD has been shown to be associated with increased risk of comorbidity with DMII, HT and CAD and it has been speculated that the chronic systemic inflammation and recurrent infections associated with COPD may be a risk factor for the development of these [10, 11]. HT has been shown to be twice as prevalent among diabetic patients than among those without diabetes, and reciprocally, hypertensive patients are more likely to develop DMII compared with normotensive persons [15]. Thus DMII and HT often coexist as comorbid conditions and serve to exacerbate each other [15]. DMII patients have been shown to have excess risk of CAD and CHF even after controlling for shared risk factors [14, 16, 17]. Furthermore, diabetic cardiomyopathy contributes significantly to CAD morbidity and mortality in diabetic patients, especially when paired with coexistent HT [15].

Limitations of our proposed model include that a log-linear relationship is assumed between disease prevalence and calendar time, and this may not be suitable for certain applications. In addition, the assumption that the shared and disease-specific spatial components are independent of each other does not allow for the possibility of interaction between unobserved underlying spatial factors. Moreover, there is a degree of ecological fallacy in generalising area-level measures of SES to all residents of each SLA. This may have obscured the true association between SES and risk of hospitalisation.

We have demonstrated the utility of our selected model in the context of a case study that examines shared spatial factors for hospital admission rates for five ambulatory-care sensitive conditions. Findings of this case study suggest that a small proportion of the geographic variation that is shared between DMII, COPD, CAD, CHF and HT is related to socio-economic status. We consider that the remainder of the shared spatial component is due to contributions from other shared risk factors. These are likely related to access to primary care and lifestyle factors, and may include distance to primary care provider, rurality, availability of fast food, healthy food and green spaces, walkability, prevalence of smoking and obesity. Further research linking the shared and disease-specific spatial variation to further covariates, such as the Accessibility/Remoteness Index of Australia (ARIA), presence of a hospital and total number of primary care providers within each region, and prevalence of the above lifestyle factors would aid interpretation. This would inform health policy decision-making to tailor interventions to factors found to be important.

In applications where the shared risk factors are a stronger predictor of one or two diseases compared with other diseases being modelled, it is expected that the shared risk component estimated would only partially reflect the spatial pattern of that particular risk factor. The excess variation in the disease with the stronger association with this risk factor, would be captured by the disease-specific component for that disease. Thus, if SES is a stronger predictor of DMII compared with other diseases, then we would expect that part of this effect would be captured in the DMII-specific spatial component. Similarly, if a putative risk factor such as prevalence of smoking is shared among all diseases modelled but is a stronger predictor of COPD compared with the other diseases, then we would expect that the COPD-specific spatial component would partially capture the spatial variation associated with this factor. More than half of all spatial variation was explained by disease-specific spatial factors across all years of this case study, reflecting varying aetiology and differential effect of risk factors for the five conditions under study.

Within the general population, admission rates were found to be highest for CAD, followed by COPD, CHF, DMII and were lowest for HT, likely reflecting the severity of acute illness for each condition. While admission rates for DMII and COPD increased over the study period, potentially reflecting the need for better quality of primary care, they decreased for CHF and remained stable for CAD and HT. After accounting for SES, the effect of the remaining underlying shared spatial factors appeared to change over time. Further research adjusting for age/gender population distributions over time and examining the association of changes in other covariates over time with temporal variation in the shared component of these diseases may be useful in explaining these variations.

In an epidemiological context, we found evidence of geographic disparity in hospitalisation rates for five ACS conditions in the region, and are able to highlight areas within NSW most at risk for hospitalisation for all five conditions, viz. those with the largest shared spatial components. These areas may benefit most from additional services for early detection, closer monitoring and management of these conditions within a primary care setting to avoid hospitalisation for complications. SLAs identified as having the largest shared underlying component for all five diseases all contain a hospital; availability of a hospital may be associated with higher hospitalisation rates for residents of these SLAs but this was not formally assessed within this study. Further study examining the association between level of primary care and hospital care provision within each area with the shared underlying risk of hospitalisation would be useful in informing health policy decisions.

The methodology used in this case study is immediately applicable to other datasets, and to any combination of diseases with shared risk factors. These could include dietary factors, lifestyle factors such as smoking, alcohol, physical activity, and access to healthcare. The model is useful to health policy planners to highlight regions with high values of the shared component for adverse health outcomes, incidence and mortality. These regions can then be targeted for suitable interventions relevant to the underlying shared risk factors.

Strengths of our case study include that we were able to analyse data from a large number of residents from a large regional area of NSW, Australia and spanning five years, allowing examination of both spatial and temporal patterns of five related conditions. Limitations of our case study include that the age of the data limits the ability for future temporal prediction. These linked hospital admissions data were made available for this study via our collaboration with the local health service. The process for obtaining access to these data changed during the course of our research and more recent data from 2006 was not covered under this revised process. Therefore, it was not possible to obtain more recent data from 2006 under our collaborative agreement. The rate of diabetes hospital admission has remained relatively stable over the past several years and so we believe our results are robust and indicative of more recent data [41].

A condition of data access in this study was that individual areas not be identified in the published work. Although we were able to discuss the area-specific results with individual Local Health Districts, these results are not available for public release. It is now possible to obtain ethics and data custodian approval to access and publicly release area-specific data. However, this process was not available to us at the time of our study.

Another limitation is that information regarding SES and ERP was available for only one year of the study. Moreover, the ERP is based on usual place of residence, thus transient populations with a usual address outside the health region were excluded from the analysis. However, the small number of admissions within the region for any transient populations are unlikely to have a substantial impact on our analysis.

Information regarding the prevalence of other underlying aetiological factors was not available at a spatial and temporal level. Therefore, it is difficult to assess whether the variability in spatial components over time is related to a change in underlying covariates or to assess the possibility of hospital data coding errors in 2004 contributing to the variation. A sensitivity analysis of the extended model using only data from 2001 to 2003 yielded similar estimates to those reported in Tables 5 and 6, but without any unexpectedly low estimates for *δ*_*jk*_ as observed for 2004 in the full model (S1 Table). There is potential for further investigation of this with more detailed data.

This case study identifies the role of SES versus other shared and disease-specific factors in explaining variation in potentially avoidable hospital admissions for five conditions, and highlights regions most at risk. By targeting these regions for the provision of better quality primary care, early detection, monitoring and management of disease, we have the potential to reduce the costs and social burdens of hospitalisation for these conditions in the future.

## Supporting information

S1 Text Measures of model comparison. (PDF)

S1 Table Estimated parameters from the selected joint disease model restricted to 2001–2003 data. Coefficients are summarised with regard to posterior mean and 95% credible interval.
(PDF)

## Abbreviations

ABS: Australian Bureau of Statistics
ACS: Ambulatory care sensitive
CAD: Coronary arterial disease
CAR: Conditional autoregressive
CHF: Congestive heart failure
CI: Credible interval
COPD: Chronic obstructive pulmonary disease
DIC: Deviance Information Criterion
DMII: Type II diabetes mellitus
ERP: Estimated resident population
GLMM: Generalised linear mixed model
HT: Hypertension
ICD10: International Statistical Classification of Diseases-10
IEO: Index of Education and Occupation
IER: Index of Economic Resources
IRSAD: Index of Relative Socio-Economic Advantage and Disadvantage
IRSD: Index of Relative Socio-Economic Disadvantage
MCMC: Markov chain Monte Carlo
NSW: New South Wales
SEIFA: Socio-Economic Indexes for Areas
SES: Socio-economic status
SLA: Statistical Local Area
