# Supplementary material for: Joint modelling of potentially avoidable hospitalisation for five diseases accounting for spatiotemporal effects: A case study in New South Wales, Australia
Source: PLoS One. 2017 Aug 30;12(8):e0183653. doi: 10.1371/journal.pone.0183653 (PMC5576724; doi:10.1371/journal.pone.0183653)
Supplement: S1 Text — (PDF) [file pone.0183653.s001.pdf]

S1 Text: Measures of model comparison

1. Deviance Information Criteria

The Deviance Information Criteria (DIC) is a sum of two components, a term that measures goodness of fit ($\overline{D}$) and a term that penalises models for the number of parameters (p_D), thus favouring more parsimonious models [1]. Here $\overline{D}$ is expected deviance over the course of MCMC, T is the total number of iterations, $D(y, \theta^{(t)})$ is the deviance of the unknown parameters of the model θ at iteration t, y are the data and $p(y, \theta^{(t)})$ is the likelihood function of observing the data given the model at iteration t.

$$DIC = \overline{D} + p_D$$
$$\overline{D} = \frac{1}{T}\sum_{t=1}^{T} D(y, \theta^{(t)})$$
$$D\left(y, \theta^{(t)}\right) = -2\log\left(p\left(y, \theta^{(t)}\right)\right)$$

A smaller value of $\overline{D}$ indicates a relatively better model fit. Smaller values of DIC are favoured overall. The comparison is relative; even if all models fit poorly, one will always correspond to the "best" as determined by the smallest DIC.

2. Log likelihood

The log likelihood (LL) for each model was calculated following each MCMC iteration, to reflect uncertainty in model parameters. The average log likelihood over all iterations is then reported, with higher values indicative of better model fit, relative to other models under consideration.

3. Root mean squared error

Replicate data for each observation y_{ijk} were generated at each iteration t based on estimated parameters of the model, and compared to observed data for each observation y_{ijk} as follows:

$$\tilde{y}_{ijk}^{rep(t)} \sim Poisson(n_i \theta_{ijk}^{(t)})$$
$$RMSE^{(t)} = \sqrt{\frac{\sum_{i=1}^{I}\sum_{j=1}^{J}\sum_{k=1}^{K}(\tilde{y}_{ijk}^{rep(t)} - y_{ijk})^2}{IJK}}$$

where $\tilde{y}_{ijk}^{rep(t)}$ is the replicate observation for each observation Y_{ijk} at iteration t. The root mean squared error (RMSE) can be used to measure the overall discrepancy between predicted and observed values. Similar to LL, the average value of RMSE over MCMC iterations is reported, with smaller values indicative of improved fit.

4. Predictive concordance

The replicate data $\tilde{y}_{ijk}^{rep(t)}$, were used to obtain the 95% CI for each observation across the T iterations. The percentage of observed values, Y_{ijk}, that fall into their corresponding 95% CI was then used as a measure of predictive concordance. Adequate model performance is inferred if 95% of observations fall within their respective interval. In general, lower

concordance suggests that the model does not fit the data well, and higher concordance may suggest overfitting [2].

References

1. Spiegelhalter D, Best NG, Carlin B, Van Der Linde A. Bayesian measures of model complexity and fit. J R Stat Soc. 2002;64(4):583-639.
2. Gelfand A. Model Determination using sampling based methods. In: Gilks WR, Richardson S, Spiegelhalter D, editors. Markov Chain Monte Carlo in Practice. Boca Raton: Chapman & Hall; 1996. p. 145-61.
