# Supplementary material for: Joint modelling of potentially avoidable hospitalisation for five diseases accounting for spatiotemporal effects: A case study in New South Wales, Australia
Source: PLoS One. 2017 Aug 30;12(8):e0183653. doi: 10.1371/journal.pone.0183653 (PMC5576724; doi:10.1371/journal.pone.0183653)
Supplement: S1 Table — Coefficients are summarised with regard to posterior mean and 95% credible interval. (PDF) [file pone.0183653.s002.pdf]

S1 Table: Estimated parameters from the selected joint disease model restricted to 2001-2003 data. Coefficients are summarised with regard to posterior mean and 95% credible interval.

Disease j	1 (DMII)	2 (COPD)	3 (CAD)	4 (HT)	5 (CHF)
α_j	-6.229 (-6.316, -6.148)	-5.856 (-5.927, -5.786)	-5.163 (-5.213, -5.114)	-7.935 (-8.149, -7.743)	-6.088 (-6.167, -6.009)
exp(α_j)*10,000	20 (18-21)	29 (27-31)	57 (54-60)	4 (3-4)	23 (21-25)
β_j	0.134 (0.061, 0.197)	0.098 (0.044, 0.152)	0.033 (-0.007, 0.069)	0.012 (-0.149, 0.159)	-0.023 (-0.082, 0.039)
σ	0.037 (0.004, 0.084)				
ω_j	0.070 (0.007, 0.153)	0.069 (0.008, 0.157)	0.038 (0.002, 0.099)	0.31 (0.142, 0.502)	0.084 (0.008, 0.160)
σ_S	0.553 (0.365, 0.814)				
σ_{Vj}	0.209 (0.065, 0.401)	0.287 (0.123, 0.495)	0.564 (0.369, 0.856)	0.822 (0.477, 1.322)	0.140 (0.010, 0.340)
ϕ	-0.007 (-0.012, -0.002)				
γ_j	0.000 (-0.002, 0.004)	0.002 (-0.001, 0.005)	0.000 (-0.004, 0.006)	-0.006 (-0.013, 0.003)	0.002 (0.000, 0.005)

DMII=diabetes mellitus type II, COPD=chronic obstructive pulmonary disease, CAD=coronary arterial disease, HT=hypertension, CHF=congestive heart failure
